# Tooth alignment and pain experience with A-NiTi versus Cu-NiTi: a randomized clinical trial

**DOI:** 10.1186/s12903-021-01789-5

**Published:** 2021-09-06

**Authors:** Fatemeh Azizi, Aida Extiari, Mohammad Moslem Imani

**Affiliations:** 1 Department of Orthodontics, Faculty of Dentistry, Kermanshah University of Medical Sciences, Kermanshah, Iran; 2 Students Research Committee, Kermanshah University of Medical Sciences, Kermanshah, Iran

**Keywords:** Orthodontic appliances, Orthodontic wires, Pain perception, Malocclusion

## Abstract

**Background:**

Nickel-titanium (NiTi) archwires are routinely used for initial leveling and alignment of teeth in orthodontic treatment. This study aimed to clinically compare the level of pain and tooth alignment in orthodontic treatment with A-NiTi versus Cu-NiTi archwires.

**Methods:**

In this parallel randomized clinical trial, 88 orthodontic patients (12–25 years) with an irregularity index > 2 mm in the anterior site of the lower dental arch who required non-extraction orthodontic treatment of the lower arch were randomized into two age- and sex-matched groups (n = 44) for treatment with A-NiTi and Cu-NiTi initial archwires. Each archwire was used for 6 weeks. After 6 weeks, the irregularity index was measured, and the level of pain was scored using the Modified McGill pain questionnaire (MPQ) and visual analog scale (VAS) according to the time of onset and duration of pain, and analgesic intake. Data were analyzed by paired *t* test, independent samples *t* test, and Chi-square test (*P* < 0.05).

**Results:**

The irregularity index significantly decreased in both groups after 6 weeks of treatment (*P* < 0.001). However, the difference in this respect was not significant between the two groups (*P* > 0.05). Pain perception (*P* = 0.487), duration of pain (*P* = 0.546), and analgesic intake (*P* = 0.102) were not significantly different between the two groups either.

**Conclusion:**

Both A-NiTi and Cu-NiTi archwires are equally effective for tooth alignment in the anterior site of the lower dental arch and have no significant difference with regard to the level of pain experienced by patients.

*Trial registration number*: IRCT20190705044102N1 and Name of the registry: Iranian registry of clinical trials (https://irct.ir/) Date of registration: September, 26, 2019

## Background

Orthodontic treatment is performed for correction of dental irregularities. Archwires generate the force required for orthodontic tooth movement. Selection of an appropriate archwire in fixed orthodontic treatment for load application to the teeth can contribute to treatment success. Initial archwires are used at the onset of fixed orthodontic treatment, and are mainly used for correction of crowding and slight tooth rotation. Light continuous force is ideal for orthodontic treatment since it would result in controlled and predictable tooth movement with minimal damage to the teeth and their supporting structures. Clinically, efficient force should cause maximum tooth movement with minimum root resorption and pain [1].

The force generated by an archwire highly depends on the physical properties of the material used for the fabrication of archwire. At present, due to the wide range of mechanical properties of different alloys, archwires with the same size and shape but different stiffness can be fabricated [1]. Ideally, an archwire should be biocompatible, must have low stiffness to release light force upon activation, high strength, and resistance to permanent deformation, should preserve its elasticity for a long period of time, and must be easy to use and low-cost [1].

Selection of a suitable archwire is an important step that determines the success of leveling and alignment of the teeth [2].

At present, different orthodontic archwires are used for the initial phase of orthodontic treatment. Nickel–titanium (NiTi) archwires are most commonly used for the initial leveling and alignment of the teeth due to the optimal elasticity, low stiffness, high flexibility, and high spring back of the NiTi alloy [2–4]. NiTi alloy is available in two crystalline phases of martensite and austenite (A-NiTi), with different physical and mechanical properties. Formation of each phase depends on the magnitude of the applied stress and temperature change. Phase transformation alters the properties of the wire without changing the type of material. Presence of both phases in an archwire results in super-elasticity of the alloy. This unique property is favorable for initial leveling and alignment of the teeth. The temperature at which, phase transformation occurs is known as the transition temperature. The A-NiTi wire, mainly composed of the austenite (high-temperature) phase, has higher elasticity than stainless steel. Bending the NiTi wire by at least 2 mm helps in formation of the martensite phase. This process is referred to as the stress‐induced martensitic transformation. To achieve maximum clinical efficacy, the transition temperature should be adjusted close or right below the oral temperature [1]. The NiTi archwires can be divided into four types according to their crystalline structure and phase transformation: stabilized (such as Nitinol, Titanal, and Orthonol), super-elastic austenite type (such as A-NiTi), thermodynamic martensite type (such as Cu-NiTi and Neo Sentalloy), and graded thermodynamic type (such as Bioforce) [1].

The A-NiTi archwire has excellent spring back compared with other archwires, and can apply light load in a wide range due to its molecular and crystalline composition [5]. Following the introduction of NiTi archwires to orthodontics, different elements were added to this alloy to confer clinical advantages. Copper (Cu) is added to NiTi alloy to decrease the loading stress, and lead to more effective orthodontic tooth movement by provision of optimal force [6]. The Cu-NiTi archwire has thermo-elastic properties due to its phase transition depending on temperature [7].

The load applied by the archwire should be able to induce tooth movement in the desired direction with minimal pain [3, 8]. It has been confirmed that orthodontic force application results in the release of pro-inflammatory cytokines and generation of inflammatory-like reactions [5]. The prostaglandins serve as a mediator for orthodontic tooth movement; however, they also enhance the transfer of painful stimuli and result in pain generation [9]. Formation of ischemic areas in the periodontal ligament with sterile necrosis can also cause orthodontic pain. Mild inflammation of the pulp and allergic reactions contribute to pain and edema in orthodontic patients as well [7].

In vitro investigations under controlled conditions have supported the unique performance of new archwires such as thermos-elastic wires [2]. The cost of Cu-NiTi archwires is about twice that of A-NiTi archwires. Considering their high cost and oral clinical conditions, their performance should be investigated in the clinical setting [5]. Adequate clinical evidence is not available on this topic, and clinical trials are required to compare the performance of thermo-elastic and super-elastic archwires [2]. Thus, this study was carried out aiming to clinically compare the rate of tooth alignment and the level of pain in orthodontic treatment with A-NiTi versus Cu-NiTi archwires.

## Methods

This parallel randomized clinical trial was conducted at the School of Dentistry of Kermanshah University of Medical Sciences in 2019. The study protocol was approved by the ethics committee of this university (IR.KUMS.REC.1398.632), and registered in the Iranian Registry of Clinical Trials (IRCT20190705044102N1) that the first registration date was 26/09/2019.

### Trial design

This parallel randomized clinical trial evaluated orthodontic patients in two groups of A-NiTi and Cu-NiTi archwires. The study was carried out and reported in accordance with the CONSORT guidelines.

### Participants, eligibility criteria, and settings

The study population was derived from patients requiring fixed orthodontic treatment of the lower arch presenting to private dental offices in Kermanshah City. Eligible patients were enrolled by convenience sampling after they willingly signed informed consent forms. The inclusion criteria were (1) requiring non-extraction fixed orthodontic treatment of the lower arch, (2) patients with complete permanent dentition (except for the second and third molars), (3) age between 12 and 25 years, (4) crowding of the anterior site of the lower dental arch, (5) irregularity index > 2 mm in the mandible, (6) not performing the expansion and distalization phase of the lower arch during the study period, (7) absence of missing or spacing in the lower arch.

The exclusion criteria were (1) presence of active periodontal disease, (2) history of previous orthodontic treatment, (3) systemic diseases affecting pain perception, (4) chronic intake of analgesics and non-steroidal anti-inflammatory drugs, (5) presence of blocked-out teeth that did not allow bracket bonding in the initial bonding session, and (6) patients with a history of toothache, mucosal ulcers, or temporomandibular disorders. A total of 88 eligible patients were enrolled.

### Interventions

Control group: After obtaining an alginate impression from the mandibular arch, and banding and bonding of the teeth with MBT0/022 system (American Orthodontics, Sheboygan, Wisconsin, USA) and standardization of bracket placement, 0.014-inch A-NiTi archwire (American Orthodontics, Sheboygan, Wisconsin, USA) was used.

Intervention group: After obtaining an alginate impression from the mandibular arch, and banding and bonding of the teeth with MBTT0/022 system (and standardization of bracket placement) similar to the control group, 0.014-inch Cu-NiTi archwire (American Orthodontics, Sheboygan, Wisconsin, USA) was used.

The archwires were placed such that they engaged all the lower teeth. Also, type of engagement with the elastomeric ligature (Dentaurum GmbH & Co., KG, Ispringen, Germany) was the same for all patients.

**Fig. 1:**
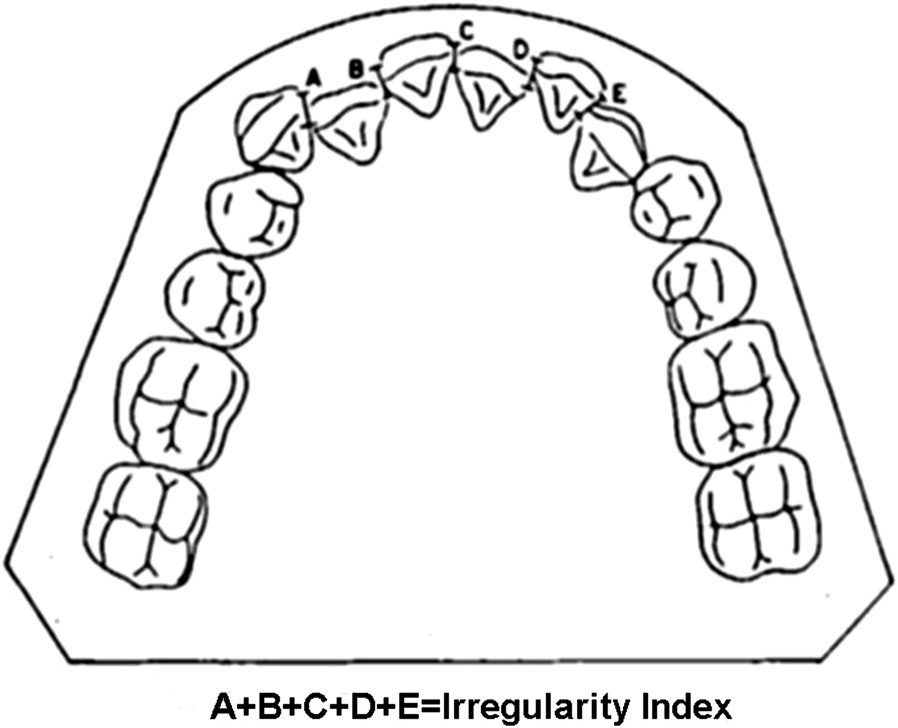
Schematic view of the calculation of irregularity index

To control for the confounding factors, bonding of the maxillary arch was not performed or at least the wire of the maxillary arch was not changed during the follow-up period (6 weeks). Also, if less than 2 months had passed since the primary orthodontic impression making, the impression was not repeated prior to the lower arch bonding, and the same primary impression was used. The patients were recalled after 6 weeks, and an alginate impression was made. The irregularity index was calculated for the anterior segment (Fig. 1), and the level of pain was determined using the modified McGill pain questionnaire (MPQ) with confirmed validity and reliability [10], and a pictorial visual analog scale (VAS). For this purpose, all patients were provided with the MPQ after archwire placement, and were instructed on how to fill it out at different times. The patients were allowed to use 325 mg acetaminophen for pain control [11], but were requested to write down the number of analgesics taken in the questionnaire.

### Outcomes (primary and secondary)

The primary objective of this study was to assess the amount of tooth alignment by the irregularity index while the secondary outcome was to assess the level of perceived pain by using the MPQ and the pictorial VAS (time of initiation of pain, duration of pain, and analgesic intake).

### Sample size calculation

The sample size was calculated to be 44 in each group according to a study by Mahmoudzadeh et al. [12] assuming α = 0.05, β = 0.2, standard deviation of 0.98, and D = 0.6

### Interim analyses and stopping guidelines

There were no interim analyses or stopping guidelines.

### Randomization

Block randomization was applied. For this purpose, four levels were defined according to gender and age range of 12–17.99 years, and 18–25 years. Allocation of samples to the groups was performed based on the abovementioned levels by using 8 random blocks. Two blocks were defined for treatment with A-NiTi and Cu-NiTi archwires. Of the two binary combinations of the two archwires, one block was randomly selected. Eventually, 22 patients were allocated to each level (4 levels, a total of 88 patients). Half of them were treated with A-NiTi and the other half with Cu-NiTi archwire.

### Blinding

The examiner who calculated the irregularity index (by assessment of dental casts), and the pain score was not aware of the group allocation of patients. After impression making, pouring the casts, and banding and bonding, the patients received the archwires based on their group allocation. The research director coded the patients’ casts. The statistician who performed the statistical analysis was also blinded to the group allocation of patients. The patients were blinded to the type of archwire they received as well.

### Statistical analysis

The Kolmogorov–Smirnov test was applied to analyze the normal distribution of the data. Comparisons were made using independent samples *t* test and paired *t* test via SPSS version 16 at 0.05 level of significance.

## Results

### Participant flow

**Fig. 2:**
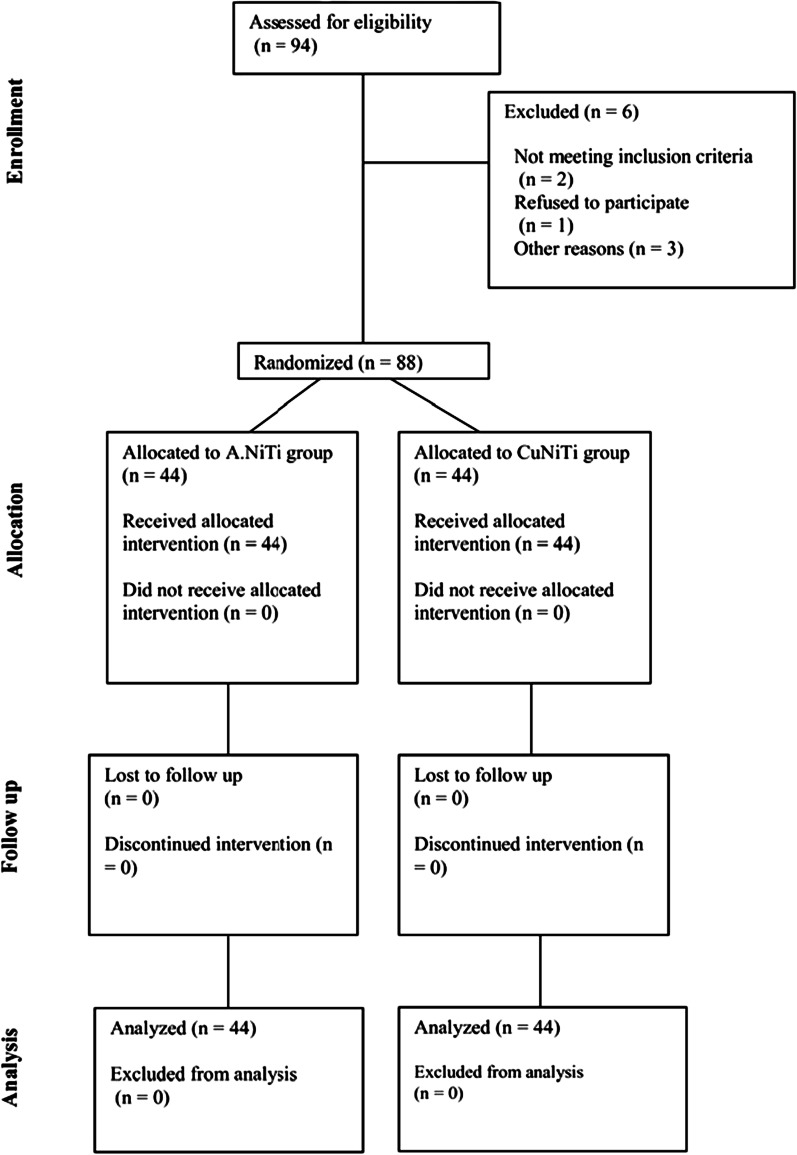
Flow diagram of the study

A total of 88 orthodontic patients were evaluated in two groups (n = 44). Table 1 presents the demographic information of patients. In each group, 22 were males and 22 were females (*P* = 1.00). The mean age of patients was 18.8 ± 4.2 years in the A-NiTi and 18.3 ± 4.7 years in the Cu-NiTi group. The two groups were not significantly different regarding age (*P* = 0.528). Figure 2 shows the flow diagram of the study.

**Table 1:** Demographic information of patients

Variable	A-NiTi (%)	Cu-NiTi (%)	*P* value	Test statistic
Gender				
Male	22 (50)	22 (50)	1*	0.001
Female	22 (50)	22 (50)		
Mean age (years)	18.8 ± 4.2	18.3 ± 4.7	0.598**	0.528
Age groups (years)				
12 ≤ age < 18	24 (48)	26 (52)	0.667*	0.185
≤ 18 age ≤ 25	20 (52.6)	18 (47.4)		

*Chi-square test

**Student *t* test

### Harms

No patients were harmed during the study.

### Subgroup analyses

#### Primary outcome

Table 2 presents the irregularity index of the two groups before and after the intervention. As shown, the mean irregularity index was almost the same in the two groups at baseline (*P* = 0.855). After the intervention, the mean irregularity index in the Cu-NiTi group was slightly higher than that in the A-NiTi group but not significantly (*P* = 0.065).

**Table 2:** Mean irregularity index (mm) in the two groups before and after treatment

Time	Study group irregularity index		*P* value*	Test statistic
	A-NiTi mean (SD)	Cu-NiTi mean (SD)		
Before treatment	8.73 (2.22)	8.34 (2.15)	0.395	0.855
After treatment	6.17 (1.82)	5.47 (1.69)	0.065	1.87
*P* value**	< 0.001	< 0.001		
Test statistic	13.17	13.78		
Change in irregularity index (mm)	2.56	2.87		

**Paired *t* test, *Independent samples* t* test

*SD* standard deviation

In both the A-NiTi and Cu-NiTi groups, the mean irregularity index after the intervention was significantly lower than that before the intervention (*P* < 0.000).

#### Secondary outcome

Table 3 compares the perceived level of pain after the intervention between the two groups. As shown, although the mean score of pain in the Cu-NiTi group was slightly higher than that in the A-NiTi group, this difference was not significant (*P* = 0.487). Also, the mean time interval between the treatment onset and initiation of pain in the Cu-NiTi group was slightly longer than that in the A-NiTi group but not significantly (*P* = 0.603).

**Table 3:** Comparison of the perceived level of pain in the two groups and the time interval between the treatment onset and initiation of pain

	Number of cases	Study group		*P* value*	Test statistic
		A-NiTi mean (SD)	Cu-NiTi mean (SD)		
Pain score	44	5.84 (2.04)	6.16 (2.23)	0.487	− 0.697
Time interval between treatment onset and pain initiation (hours)	43	5.45 (6.31)	6.14 (5.93)	0.603	− 0.521

*One sample *t* test

*SD* standard deviation

Table 4 presents the pain duration in the two groups. As shown, the two groups were not significantly different in this respect. Table 5 presents the frequency of analgesic intake by the patients in the two groups. Although the frequency of analgesic intake was higher in the A-NiTi group, this difference was not statistically significant (*P* = 0.102).

**Table 4:** Duration of pain in the two groups

Group	One day	Two days	Three days	Four days	More than 4 days	*P* value*
A-NiTi (%)	2 (4.5)	8 (18.2)	13 (29.5)	11 (25)	10 (22.7)	0.546
Cu-NiTi (%)	6 (13.9)	9 (20.9)	13 (30.2)	7 (16.3)	8 (18.6)	

*Fisher’s exact test

**Table 5:** Frequency of analgesic intake by the patients in the two groups

Group	Analgesic intake		*P* value	Test statistic
	Yes (%)	No (%)		
A-NiTi (%)	15 (34.1)	29 (65.9)	0.102	2.682
Cu-NiTi (%)	8 (18.6)	35 (81.4)		
Total	23 (26.4)	64 (73.56)		

## Discussion

This parallel randomized clinical trial compared A-NiTi super-elastic archwire and Cu-NiTi thermo-elastic archwire regarding the amount of tooth alignment and the level of pain experienced by patients in this process. The results showed no significant difference between the two initial archwires in their efficacy, or level of perceived pain and number of analgesics taken by patients. The results also showed that the mean irregularity index was the same in the two groups at baseline, and significantly decreased following the intervention in both groups. This finding indicates that both archwires were equally effective for correction of crowding of the anterior site of the lower dental arch.

In the present study, the A-NiTi archwire was compared with the Cu-NiTi archwire. Addition of copper to NiTi decreases the loading stress and provides a relatively high unloading stress, which can lead to more efficient orthodontic tooth movement [13]. Nonetheless, the current results revealed equal efficacy of the two archwires in correction of crowding of the anterior site of the lower dental arch. In line with our findings, Aydin et al. [14] Gok et al. [15] and Pandis et al. [3] demonstrated that Cu-NiTi archwire did not have higher efficacy than NiTi archwire for leveling and alignment of the mandibular teeth. Also, Atik et al. [6] compared Tanzo Cu-NiTi and NT3 super-elastic NiTi (SENT) archwires and found no significant difference between them in correction of crowding of the maxillary teeth. Similarly, Nabbat and Yassir [16] reported equal efficacy of heat-activated NiTi (HANT) archwire and SENT archwire for alignment of the teeth. Sebastian et al. [17] reported that coaxial tubular and single-stranded SENT archwires were equally effective for correction of crowding of the anterior site of the lower dental arch. Moreover, Nordstrom et al. [18] demonstrated equal efficacy of TiNbTaZr and the conventional NiTi archwires for alignment of the teeth. Mahmoudzadeh et al. [12] clinically compared A-NiTi and HANT archwires for alignment of the teeth and reported no significant difference in the irregularity index between the two groups. Ulhaq et al. [19] assessed the BioCosmetic, Titanol, TP Aesthetic, and Tooth Tone archwires and found no significant difference between them. Abdelrahaman et al. [20] reported no significant difference between SENT, conventional NiTi and thermos-elastic NiTi archwires in alignment of the teeth.

In contrast to the present study and the aforementioned ones, Maria de Castro Serafim et al. [11] found that HANT archwire was significantly faster than the conventional NiTi archwire in leveling and alignment of the teeth. Also, according to Sebastian [21] the coaxial SENT archwire was significantly superior to single-stranded SENT archwire in terms of clinical efficacy. Difference between their results and ours may be due to the use of different types of archwires.

The current study showed 2.56 mm and 2.87 mm reduction in the irregularity index in the A-NiTi and Cu-NiTi groups, respectively. Nabbat and Yassir [16] in a study with a relatively similar methodology to ours reported 2.69 mm and 2.74 mm reduction in the irregularity index in use of HANT and SENT archwires, respectively, which were close to the values reported in our study.

In contrast to the present study, Sebastian et al. [17] reported 6.17 mm and 4.88 mm reduction in the irregularity index in the anterior site of the lower dental arch by using coaxial tubular SENT and single-stranded NiTi archwires, respectively, which were considerably greater than the values reported in the present study. It appears that lower mean age in their study may be an important factor explaining the difference between their results and ours, because it has been demonstrated that age significantly affects the success of orthodontic tooth movement [22]. Cappellette et al. [23] reported that orthodontic traction of teeth into the dental arch in younger patients has a higher success rate than in older patients. Orton et al. [24] found that the duration of treatment would be longer after the completion of the pubertal period. Also, Ulhaq et al. [19] reported that the reduction in the irregularity index was 3.86 mm in use of BioCosmetic archwire, 4.51 mm in use of Titanol, 4.13 mm in use of TP Aesthetic, and 4.21 mm in use of Tooth Tone archwire. Greater rate of reduction in the irregularity index in their study compared with ours may be attributed to longer treatment period in their study, which was 63 days.

The current results showed no significant difference between the Cu-NiTi and A-NiTi groups in pain parameters (mean pain score, time interval between the onset of treatment and initiation of pain, and duration of pain). Since there was no significant difference between the two groups in the number of taken analgesics, similarity of the pain parameters in the two groups can be attributed to the equality of the two archwires regarding pain generation, and ensures absence of significant effect of analgesic intake as a confounder on the results. No significant difference in the level of pain experienced by patients in the two groups of archwires was in agreement with the findings of Nabbat and Yassir [16] comparing HANT and SENT, Mahmoudzadeh et al. [12] comparing A-NiTi and HANT, Abdelrahman et al. [8] comparing SENT, conventional NiTi, and thermos-elastic NiTi, and Sandhu and Sandhu [25] comparing SENT and multi-stranded stainless steel archwires. Unlike our study, Cioffi et al. [26] revealed that the level of pain at 2, 3 and 4 days following the installation of HANT archwire was significantly lower than that in use of SENT archwire. This controversy may be due to the different methodologies and use of various types of archwires.

In the present study, the two groups were matched in terms of gender and mean age of patients, with no significant difference, which was strength of this study and increased the reliability of the results. Moreover, blinding was performed to minimize bias, which further added to the accuracy of the results.

Reporting results after a short period of follow-up (6 weeks) was a limitation of this study. However, studies with longer follow-ups to assess the long-term effects of A-NiTi and Cu-NiTi archwires are not ethical due to prolongation of the treatment course. Also, other effects of archwires such as root resorption are important and should be addressed in future studies.

## Conclusion

Both A-NiTi and Cu-NiTi archwires are equally effective for tooth alignment in the anterior site of the lower dental arch and have no significant difference with regard to the level of pain experienced by patients.

## Data Availability

The datasets used and/or analyzed during the current study are available from the corresponding author on reasonable request.

## Abbreviations

NiTi: Nickel–titanium
MPQ: McGill pain questionnaire
VAS: Visual analog scale
A-NiTi: Austenite Nickel–titanium
Cu: Copper
HANT: Heat-activated NiTi
SENT: Super-elastic NiTi
